# Ex Vivo Biosafety and Efficacy Assessment of Advanced Chlorin e6 Nanoemulsions as a Drug Delivery System for Photodynamic Antitumoral Application

**DOI:** 10.3390/molecules30030544

**Published:** 2025-01-25

**Authors:** Stéphanie Rochetti do Amaral, Mariza Aires-Fernandes, Felipe Falcão Haddad, Ana Luísa Rodriguez Gini, Cauê Benito Scarim, Fernando Lucas Primo

**Affiliations:** 1 Department of Bioprocess and Biotechnology Engineering, School of Pharmaceutical Sciences, São Paulo State University (UNESP), Araraquara 14800-903, SP, Brazil; mariza.aires@unesp.br; 2 Department of Drugs and Medicines, School of Pharmaceutical Sciences, São Paulo State University (UNESP), Araraquara 14800-903, SP, Brazil; felipe.haddad@unesp.br (F.F.H.); ana.gini@unesp.br (A.L.R.G.); caue.scarim@unesp.br (C.B.S.)

**Keywords:** nanotechnology, chlorin e6, HET-CAM, antitumoral

## Abstract

The photosensitizer (PS) in the Photodynamic Therapy (PDT) field represents a key factor, being directly connected to the therapeutic efficacy of the process. Chlorin e6 is a second-generation photosensitizer, approved by the FDA with the most desired clinical properties for PDT applications, presenting high reactive oxygen species (ROS) generation and proven anticancer properties. However, hydrophobicity is a major limitation, leading to poor biodistribution. To overcome this condition, the present work developed an up-to-date nanoemulsion incorporating Ce6 in a new nanosystem (Ce6/NE). A comprehensive study of physicochemical properties, stability, fluorescence characteristics, the in vitro release profile, in vivo and ex vivo biocompatibility, and ex vivo efficacy was established. The nanoemulsions showed the desired particle size and stability over six months, with no spectroscopic or photophysical alterations. Uptake studies demonstrated the internalization of the Ce6/NE in monolayers, with biocompatibility at the lowest concentrations. The HET-CAM assay, however, revealed a higher biocompatibility range, also indicating Ce6/NE’s potential for cancer treatment through antiangiogenic studies. These findings highlight the use of a new promising photosensitizer for PDT modulated with nanotechnology that promotes low toxicity, higher bioavailability, and site-specific delivery.

## 1. Introduction

Chlorin e6 (Ce6) is a widely studied photosensitizer recognized for its significant potential in Photodynamic Therapy (PDT), a cutting-edge treatment for cancer and various infectious diseases. It consists of a second-generation photosensitizer, derived from chlorophyll, and presents unique photophysical and photochemical properties that have encouraged extensive research and interest in clinical applications [1]. Ce6’s strong absorption in the near-infrared region, approximately 660–670 nm, enables deeper tissue penetration and efficient light activation, distinguishing it from the first-generation photosensitizers that face limitations due to their less effective light absorption and higher risks of phototoxicity [2,3].

Ce6 exhibits therapeutic efficacy in PDT through its capacity to produce reactive oxygen species (ROS), specifically singlet oxygen (^1^O_2_), when light is activated in the presence of molecular oxygen [4]. The production of ROS is a key mechanism for identifying and eliminating tumor cells via cell death, causing significant damage to cellular structures [5]. Additionally, Ce6 exhibits high singlet oxygen generation efficiency and a high molar extinction coefficient, further enhancing its potential in photodynamic performance [6].

Regarding Ce6 applications, it has been used extensively in the treatment of various cancers, being clinically approved by the Food and Drug Administration (FDA) as Talaporfin Sodium for the treatment of lung cancer and malignant gliomas [2,7]. The ability to selectively activate Ce6-based PDT at tumor sites through targeted light exposure enhances treatment outcomes while reducing systemic side effects, offering patients a minimally invasive and highly precise cancer treatment option [1,8]. Despite these promising attributes, the clinical use of Ce6 faces significant challenges, such as phototoxicity, suboptimal biodistribution, and limitations related to hydrophobicity, leading to low interaction in target tissues and being eliminated from the bloodstream rapidly [9].

Recent breakthroughs in nanotechnology, particularly the development of nanoemulsions, have provided innovative solutions to overcome limitations in the delivery and effectiveness of hydrophobic drugs such as Ce6, which are poorly delivered in biological systems, resulting in low stability, reduced internalization, and a high number of side effects [9]. Among the main colloidal nanocarriers with potential applications, polymeric and emulsified systems such as nanoemulsions stand out. Due to their small droplet size, typically ranging from 20 to 200 nm, nanoemulsions enhance the solubility and stability of hydrophobic drugs, facilitating effective cellular uptake and tissue distribution, enabling controlled and sustained release, and resulting in a targeted therapeutic effect while minimizing systemic exposure and associated side effects [10]. Regarding cancer treatment, nanoemulsions can provide effective therapeutic effects, such as significant surface area, target delivery, and rapid accumulation in vascularized tissues, being able to easily cross barriers because of their small size [11].

In the context of PDT, nanoemulsions work as efficient nanocarriers for Chlorin e6, improving its pharmacokinetic profile and enabling targeted drug delivery [12]. Through the enhanced permeability and retention (EPR) effect, these nanoformulations can accumulate in tumor tissues, providing higher therapeutic concentrations at the disease site and reducing off-target toxicity [13].

The present article focuses on the integration of Chlorin e6-loaded nanoemulsions as drug delivery systems, emphasizing how these advanced nanoemulsions address current challenges in biosafety and effectiveness using in vitro and ex vivo models. Furthermore, it highlights the potential of nanoemulsions to innovate the delivery and therapeutic performance of Ce6, paving the way for more effective applications for skin cancer treatment.

## 2. Results

### 2.1. Physical–Chemical Analysis: Particle Size, PdI, and Zeta Potential

A DLS analysis was used to obtain information about the size distribution, polydispersity index, and zeta potential of the nanoemulsions encapsulated with Chlorin e6 and the unloaded ones for 180 days. The results, expressed as the mean ± standard deviation in Table 1, indicate a narrow size distribution, with an average hydrodynamic diameter of 62.07 nm and 59.74 nm for the Ce6/NE and unloaded/NE, respectively. The PdI shows a low distribution of size populations, with values of 0.178 and 0.185 (Ce6/NE and Unloaded/NE, respectively). The zeta potential analysis showed negative values for the surface, with stable values during the time describing the characteristics connected to the surface charge of the particles [14].

These studies are fundamental for pharmaceutical and industrial applications of nanomaterials [15], and as is possible to see, the nanoemulsions developed showed a monodisperse system with a low tendency to polydispersity and aggregation. A statistical significance analysis was carried out demonstrating no statistical significance between the Ce6/NE and unloaded/NE in all parameters (particle size, PdI, and zeta potential).

The percentage of photosensitizer associated with the nanoemulsions was assessed using the calibration curve previously designed for Ce6 in DMSO through an analytical method using the ratio between the absorbance and the sample concentration, resulting in a linear equation: y = 0.0627x + 0.0128, R^2^ = 0.999, n = 3. Through the linear equation, it was possible to obtain the real concentration (Rc) of Ce6 and following the Equation (2) described in Section 4.1. The association efficiency was calculated, resulting in 79% of Ce6 associated to the system. A similar result was found for Ce6 in association with another PS [16].

### 2.2. Atomic Force Microscopy

**Figure 1:**
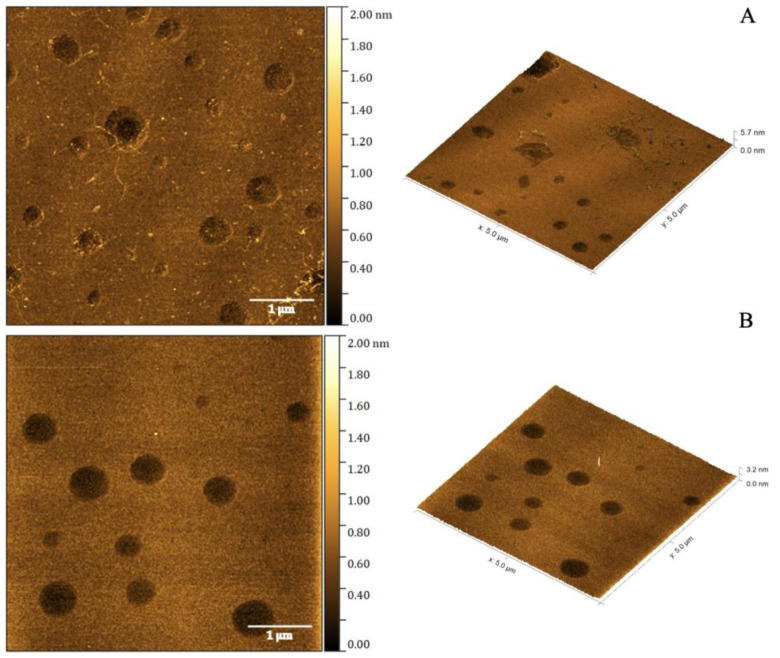
AFM images of Ce6/NE (**A**) and unloaded/NE (**B**).

Atomic force microscopy (AFM) is a well-established interface characterization method for nanoemulsions, informing on the surface topography at submicrometric and atomic and nanoresolution, as well as showing accurate images of macro/nanostructured surface topography [17]. The morphologies of the Ce6/NE and the unloaded/NE were assessed using AFM analysis, as shown in Figure 1. They appeared as a homogenous population with a spherical shape and a particle size around 50 nm, which was expected. These results confirm the formation of nanostructures, matching the theorized morphology and behaving like a colloidal system with reservoir-type characteristics.

### 2.3. Fluorescence Characterization of Chlorin e6 Nanoemulsions

#### 2.3.1. Two-Dimensional and Three-Dimensional Fluorescence Spectroscopy

**Figure 2:**
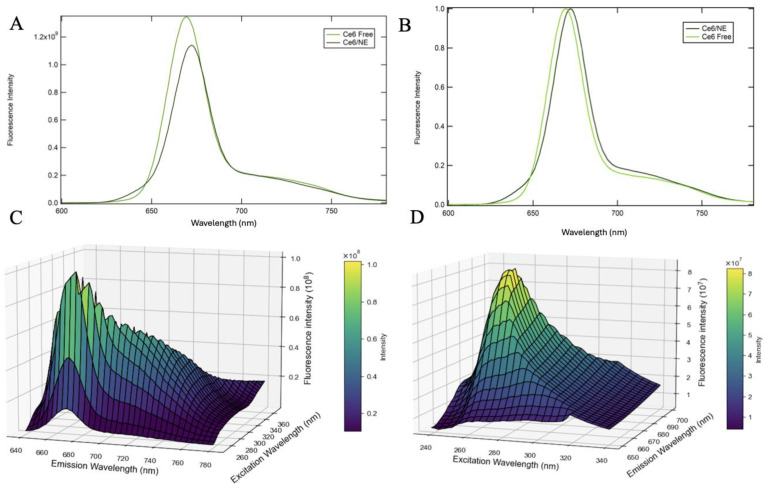
UV-Vis 2D fluorescence spectra of Chlorin e6 in dimethyl sulfoxide (DMSO) as the polar aprotic solvent (green) and Ce6/NE in water (gray), with a maximum for the Soret band (**A**) at 669 nm and 672 nm (spectral slits 10 nm/10 nm) (**A**) and a normalized version (**B**). UV-Vis 3D fluorescence spectra of Chlorin e6 in dimethyl sulfoxide (DMSO) as the polar aprotic solvent (**C**), with an emission range wavelength between 640 and 780 nm and an excitation range wavelength between 250 and 360 nm (spectral slits 10 nm/10 nm) and Ce6/NE in water (**D**) with an emission range wavelength between 650 and 700 nm and an excitation range wavelength between 238 and 340 nm (spectral slits 10 nm/10 nm).

Chlorin e6 is a second-generation porphyrin photosensitizer. It presents a characteristic absorbance value at the Soret band maximum at 405 nm and Q-band maximum at 664 nm, matching the porphyrin reported profile [18]. The 2D fluorescence studies excited the free Ce6 in DMSO and the Ce6/NE in the Soret band, both at a concentration of 8.0 µg·mL^−1^. The data presented in Figure 2 show a consistent profile when compared with free Chlorin and Ce6/NE, with insignificant changes in the maximum fluorescence intensity values.

In contrast to other spectral methods, fluorescence has demonstrated notable advantages in terms of ease of use, speed, and high sensitivity. Three-dimensional fluorescence spectroscopy, which measures emission spectra across a range of excitation wavelengths, enables the identification of samples and emerges as a potent tool for substance classification and identification [19,20].

#### 2.3.2. Fluorescence Quantum Yield

The fluorescence quantum yield (Φ_f_) is a direct measure of the effectiveness of photon absorption to photon emission conversion through fluorescence. The fluorescence quantum yield for transparent samples can be determined with the relative method, as described by WÜRTH, et al. [21]. In brief, it compares the integral emission spectra of the sample with an appropriate pre-defined standard. Obtained were values for Φ_f_ for the Ce6 in DMSO and the Ce6/NE, calculated relative to the standard (Ce6 in PBS 7.4 [22]). The results are shown in Table 2.

### 2.4. In Vitro Release Study

**Figure 3:**
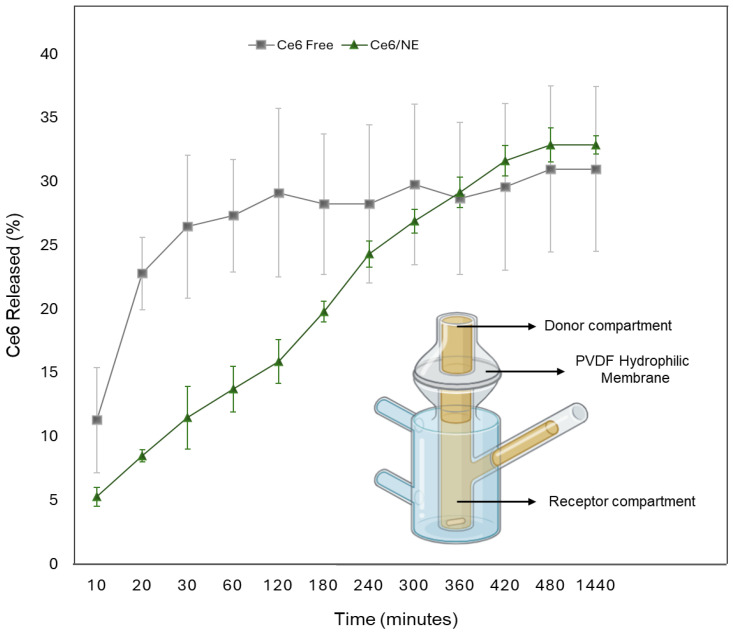
In vitro release profile of free Chlorin e6 (gray) and Ce6/NE in hydrogel polymer chain (green) within 24 h.

Prior to the in vitro release study, a solubility test was performed in four different types of release media (PBS 6.4; PBS 6.4–15% Ethanol–2% Tween 80; PBS 6.4–10% DMSO–2% Tween 80; PBS 6.4–10% DMSO–5% Tween 80), as described in Section 4.5.2. The release medium is a key part of the study, connecting with the sink conditions, ensuring that the desired amount of drug will be dissolved in the system [23]. Ce6 was practically insoluble in PBS 6.4, which can be explained by its low solubility in water. Conversely, PBS 6.4–15% Ethanol–2% Tween 80 was discovered as the most suitable for the experiment, as described by Jain et al. 2023 [16]. Sink conditions were established based on Ce6 solubility in the release medium (286.05–28.60 µg·mL^−1^). Ce6 release quantification was assessed using the calibration curve developed using a spectrofluorometer by the equation y = 1 × 10^6^ – 126,785, R^2^ = 0.9974. Figure 3 presents the in vitro release profile of the free Ce6 in the release media and the Ce6/NE thermoreversible hydrogels during 24 h, showing significant differences between the two profiles.

The release kinetics were also calculated (Table 3). Six conventional release models, Weibull, Korsmeyer–Peppas, Baker and Lonsdale, Higuchi, First Order, and Hixon and Crowell were fitted, and a regression analysis was performed. The global adjustment was made over the first 24 h, and a secondary local adjustment was made for the first 360 min (up to the inflection point).

In the global adjustment, both the free Ce6 and Ce6/NE fit the Weibull mathematical model, describing a complex release mechanism, with b being a constant describing the kinetics, where if b < 1, it represents a parabolic release mechanism, if b = 1, it is an exponential mechanism, and if b > 1, it is a sigmoidal mechanism, with an ascending curvature [24]. However, when the data until the inflexion point were analyzed, it is possible to see the difference between the release mechanisms, with the free Ce6 fitting the Weibull model and the Ce6/NE fitting Korsmeyer–Peppas in a Fickian diffusion (n < 0.5), showing diffusion as the primary transport mechanism [25]. These results support the prospect of using the formulation as a topical and/or transdermic delivery system focused on skin tumors.

### 2.5. Cellular Uptake and Cytotoxicity Assay

**Figure 4:**
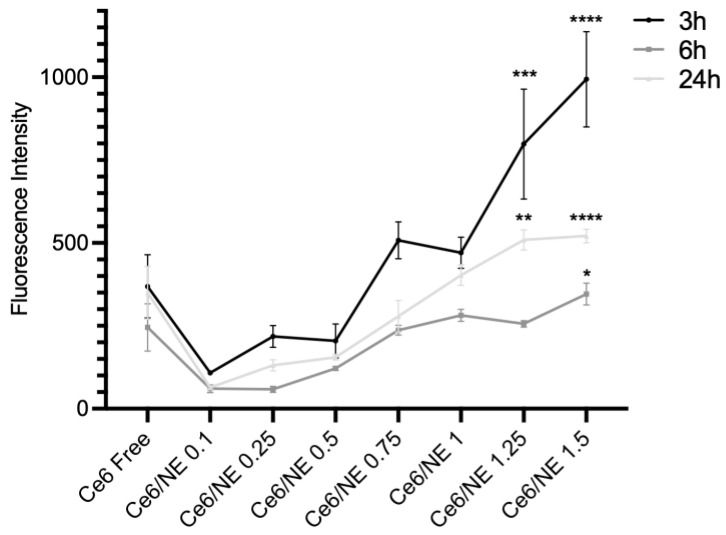
Cellular uptake assay in HDFn cells (HDFn-ATCC^TM^ PCS-201-010™, Manassas, VA, USA) of Ce6/NE 0.1–1.5 µg·mL^−1^ and free Ce6 0.5 µg·mL^−1^. Cells were incubated at different times: 3 h (black), 6 h (grey), and 24 h (light gray). Statistical significance was determined using the one-way ANOVA test followed by the Tukey post-test for multiple comparisons, comparing free Ce6 and Ce/NE. Data are presented as mean ± SEM (* *p* < 0.05; ** p = 0.001; *** p = 0.0001; **** p < 0.0001).

In order to determine the internalization of the Ce6/NE and free Ce6, a cell uptake study was developed using Neonatal Human Dermal Fibroblast (HDFn) cells in three different incubation times (3, 6, and 24 h), with concentrations ranging from 0.1 µg·mL^−1^ to 1.5 µg·mL^−1^. Higher internalization was observed for the Ce6/NE at 3 h, exhibiting the intended outcome of the nanocarrier’s active enhanced cytoplasmic internalization (Figure 4). The 6 h and 24 h incubation times presented lower intensities due to a possible intracellular metabolization process. However, there were still considerable intensities of the internalized dye.

**Figure 5:**
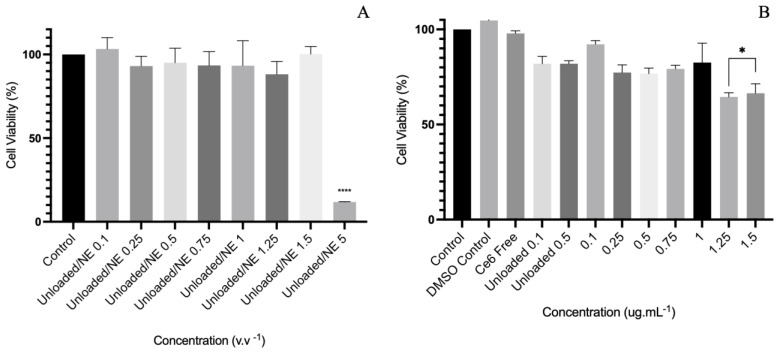
In vitro cytotoxicity assay in HDFn cells (ATCC^®^ PCS-201-010™) of unloaded/NE 0.1–5.0% *v*.*v*^−1^ (**A**); Ce6/NE 0.1–1.5 µg·mL^−1^ and free Ce6 0.5 µg·mL^−1^ (**B**). All cells were incubated for 3 h with post-application of the resazurin test. Statistical significance was determined using the one-way ANOVA test followed by the Tukey post-test for multiple comparisons. Data are presented as mean ± SEM (* *p* < 0.005; **** *p* < 0.0001).

In the sequence (Figure 5), the cytotoxicity was obtained though the classical resazurin method, as described in Section 4.8. The assay with HDFn cells was developed as a preliminary test assessing the cytotoxicity of the Ce6/NE, the free Ce6 in 0.25% DMSO, and the unloaded form, only with surfactants and water. The viability of the cells was determined considering the percentage of the control compared with the sample. The one-way ANOVA result is presented in Figure 5A for the Ce6/NE and free Ce6 and in Figure 5B for the unloaded/NE.

### 2.6. Ex Vivo Evaluation of the Biocompatibility and Antiangiogenic Activity of Ce6/NE Using the Hen’s Egg Chorioallantoic Membrane Assay (HET-CAM)

#### 2.6.1. Biocompatibility Assay

**Figure 6:**
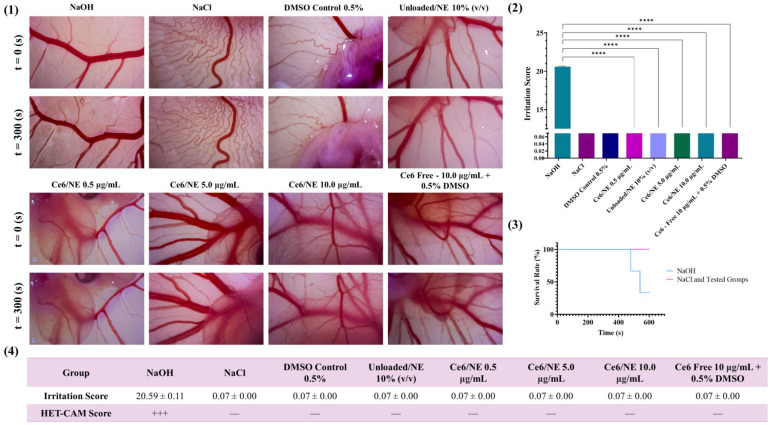
Visual representations of CAM blood vessels before (t = 0 s) and after 300 s (t = 300 s) following the application of the tested compounds (**1**). Graph showing the mean irritation scores and statistical analysis obtained from the HET-CAM assay. **** *p* < 0.0001 (**2**). Embryo survival curve after 600 s post-application of the samples (**3**). Table presenting the mean irritation score (±standard deviation) and the HET-CAM score for each experimental group (**4**), n = 3.

To assess the biocompatibility of the Ce6/NE, unloaded/NE and free Ce6 were applied in ex vivo models. The following detailed observations revealed distinct differences in vascular responses between the groups, as shown in Figure 6. The application of 0.1 M NaOH to the chorioallantoic membrane (CAM) resulted in immediate and severe hemorrhage, followed by vascular lysis and coagulation. The mean irritation score for NaOH was 20.59 ± 0.11, confirming its highly irritating and damaging nature. The statistical analysis revealed that the irritation caused by NaOH was significantly greater (*p* < 0.0001) compared to the other groups. In contrast, the administration of NaCl did not induce any observable response during the 300 s observation period, resulting in a mean irritation score of 0.07, indicating the absence of irritation. The statistical difference between the NaCl and NaOH groups was highly significant (*p* < 0.0001), confirming the non-irritating nature of NaCl.

For the groups treated with 0.5% DMSO, unloaded/NE 10% (*v*/*v*), and Ce6/NE at concentrations of 0.5 µg·mL^−1^, 5.0 µg·mL^−1^, and 10.0 µg·mL^−1^, as well as free Ce6 at 10.0 µg·mL^−1^ + 0.5% DMSO, a similarly significant difference was observed when compared to the positive control (NaOH), as none of these groups exhibited any signs of irritation (*p* < 0.0001). The HET-CAM score for NaOH was (+++), while all other groups scored (−). Additionally, the survival rate of embryos in the negative control group (NaCl) and all other experimental groups was 100%, whereas in the positive control group (NaOH), two out of three embryos died within 10 min after application.

#### 2.6.2. Antitumoral Efficacy: Antiangiogenic Activity

Angiogenesis is an essential process for the growth and dissemination of solid tumors, as the formation of a consistent blood supply is indispensable for tumor survival and expansion. Therefore, angiogenesis plays a critical role in both the initiation and progression of tumors [26,27]. The HET-CAM model, widely used due to its rapid vascular development and methodological simplicity, has proven to be an effective and accessible tool for evaluating compounds with the potential to modulate angiogenesis, acting as either inhibitors or stimulators [26,28].

**Figure 7:**
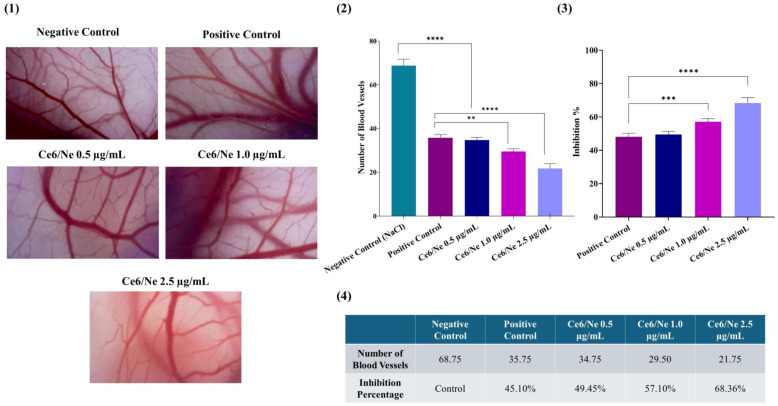
Antitumoral efficacy based on antiangiogenic potential studies. (**1**) Representative images of blood vessels observed in the chorioallantoic membrane (CAM) after treatment with the negative control, positive control, and different concentrations of Ce6/NE. (**2**) Graphical representation of the average number of blood vessels counted in the different experimental groups. (**3**) Graphical representation of the percentage of blood vessel inhibition compared to the negative control group. (**4**) Summary of the blood vessel counts and inhibition percentages. Statistical significance is indicated as follows: ** *p* = 0.0033; *** *p* < 0.0007; **** *p* < 0.0001, n = 4.

In this study, the antiangiogenic effect of Ce6/Ne was evaluated at three concentrations (0.5, 1.0, and 2.5 µg·mL^−1^) and compared to the negative control (0.9% NaCl) and positive control (methylene blue), as is shown in Figure 7. The qualitative analysis of the number of blood vessels demonstrated statistically significant differences between the negative control and all groups treated with Ce6/NE (*p* < 0.0001). The negative control showed an average of 68.75 vessels, while the positive control exhibited 35.75 vessels. The groups treated with Ce6/Ne showed averages of 34.75 vessels (0.5 µg·mL^−1^), 29.5 vessels (1.0 µg·mL^−1^), and 21.75 vessels (2.5 µg·mL^−1^). Among the concentrations tested, the groups treated with Ce6/Ne at 1.0 µg·mL^−1^ and 2.5 µg·mL^−1^ stood out compared to methylene blue, with significance values of *p* = 0.0033 and *p* < 0.0001, respectively.

The analysis of the percentage of angiogenesis inhibition revealed that methylene blue reduced the formation of new vessels by 45.10%, while Ce6/NE demonstrated increasing inhibition with higher concentrations: 49.45% (0.5 µg·mL^−1^, *p* = 0.8173), 57.10% (1.0 µg·mL^−1^, *p* = 0.0007), and 68.36% (2.5 µg·mL^−1^, *p* < 0.0001).

## 3. Discussion

Features of nanomaterials, such as particle size, the polydispersity index, and zeta potential, are crucial for pharmaceutical applications since these properties directly affect the biological environment and the impacts of toxicity, cellular absorption, and permeability [29]. Nanoemulsions can be used as a medical delivery method, presenting a small droplet size (20–200 nm) that contributes to a substantial interfacial area for drug dissolution that enhances the bioavailability and solubility of medications with low water solubility [10]. During the 180-day post-synthesis analysis period, the Ce6/NE and unloaded/NE remained in the stability range, with particle sizes between 50 and 80 nm for both formulations with no statistically significant difference between the two groups (*p* value = 0.24), PdI values between 0.1 and 0.2, and showing low tendency for agglomeration or aggregation, being a homogeneous and monodisperse system [30]. The zeta potential values remained within the proposed stability range within the analysis period (+30 mV, −30 mV), indicating surface properties that impact the biological performance and safety of the nanomaterial. In addition, the Ce6 presented high rates of association in the Ce6/NE (79%) nanosystem, showing the capacity to associate within the oily core of the NE beyond the real concentration of the PS. These qualities determine the nanomaterial’s biodistribution and unique stability, which in turn indicate the release of the medication into biological targets [14]. The AFM analysis revealed important properties from the developed nanoemulsions (Ce6/NE and unloaded/NE), confirming the average particle size, obtained from the DLS, and complemented with topography characteristics, highlighting the spherical morphology and the presence of homogeneous populations of particles [17]. Comparing AFM with the traditional methods of nanoemulsion characterizations, transmission electron microscopy (TEM) and scanning electron microscopy (SEM), it is possible to overcome limitations related to sample preparation that may result in blurred images and the destruction of droplets [31].

The fluorescence properties as 2D and 3D spectrum profiles presented no significant changes in the fluorescence profiles of the free Ce6 and Ce6/NE, showing the desired profile of Ce6. These results contribute to the use of Ce6/NE in Photodynamic Therapy (PDT), as the Q-band is the region of the spectrum for performing the therapy, where there is no competition with other photodynamic processes [18]. The desirable characteristics of a photosensitizer applied to PDT consist of strong absorption in the phototherapeutic window (650–800 nm), easy scale-up, high chemical purity, long shelf-life, low photodegradation quantum yield, low skin photosensitivity, and low dark toxicity. In this scenario, the fluorophores must present high fluorescence quantum yields, and the photosensitizers must present triplet states with long lifetimes and with high triplet quantum yields, as the PDT effectiveness is related to the generation of reactive oxygen species (ROS) by energy or to electron transfer from the excited photosensitizer to molecular oxygen [32]. The Φ_f_ data (Table 2) show values of 0.16 for the Ce6/NE and 0.36 for the Ce6 in DMSO, compared with the standard value of 0.18 [22]. The value for the nanomaterial shows maintenance of the photophysical properties, with a slight decrease when compared to the standard value (0.18 to 0.16). This increases the probability of electronic transition occurring in the formation of molecules in the triplet state: something highly favorable for its use in PDT. Analyzing Ce6 in DMSO shows how the chemical environment influences spectroscopy studies due to the organic solvents enabling modifications to the distribution of dye molecules’ electronic states upon excitation [33].

Following the breakthrough properties of nanomaterials for drug delivery, the in vitro release provides information on whether the substance can diffuse through a barrier. Considering topical applications, the use of hydrogels is part of a strategy for the vectorization of a drug across the skin, providing direct application in specific targeted area, also enhancing its stability properties by increasing viscosity and optimizing therapeutic effectiveness [34,35]. The release profile of Ce6/NE thermoreversible hydrogels using a Franz diffusion cell [36] presented in Figure 3 indicates a controlled and sustained drug release, reaching 32.9% in 24 h. In contrast, the release profile of Ce6 in the receptor solution (PBS 6.4–15% Ethanol–2% Tween 80) demonstrated a rapid release within the first 2 h (29%) but ultimately achieved 31% in 24 h. Combined, these results emphasize the potential for the modulation of drug release through the use of nanoemulsions, ensuring prolonged therapeutic concentrations and reducing concentration peaks that can lead to toxicity, contrasting with the rapid dissolution of free active ingredients, imitating their efficacy and administration [37,38,39].

The release kinetics were evaluated using mathematical models (Table 3). The adjustment was separated (global and inflexion point adjustment) to provide the closest information to the differences observed in the release profile. The Weibull model in the global adjustment fitted both systems (Ce6/NE/H and free Ce6); however, it did not show the fundamental differences in the release mechanisms, especially before the inflection point (360 min). The model still shows a complex release when compared with First-Order kinetics, for example, where the mechanism is led only by concentration changes [24]. Following the kinetics observed in the second adjustment, the Korsmeyer–Peppas model was considered the most appropriate for Ce6/NE thermoreversible hydrogels, presenting the determination coefficient (R^2^) values closest to 1.0. The fit to the Korsmeyer–Peppas model in the first phase of the release profile, with n = 0.43, suggests a release mechanism in a Fickian diffusion, leading to conditions of a time-dependent matrix, prolonging the drug release and ensuring improved kinetic control [25,40]. These results support the prospect of using the formulation as a topical and/or transdermic delivery system, focused on skin tumors.

The cell uptake and the subcellular distribution of photosensitizers and nanomaterials are extremely important, determining the effectiveness of the process. In this instance, a few variables, including the size of the material, concentration, pharmaceutical form, surface area, and chemical structure, are extremely important [41,42]. The cellular uptake of the Ce6/NE in HDFn cells was further confirmed by a fluorescence analysis (Figure 4), showing higher internalization for the Ce6/NE when compared to the free Ce6, with a specific increase in cell permeation with the increase in the concentrations. Also, within the first 3 h of internalization, it is possible to observe the best internalization rates compared to 6 h and 24 h. Cellular internalization of Chlorin e6 derivatives was previously described [43] with evaluations conducted at 1.5, 2, 3, 4, and 6 h, highlighting the optimal incubation time between 3 and 4 h depending on the Chlorin, in line with the results observed for the internalization of the Ce6/NE.

Cytotoxicity studies are crucial for drug development and the synthesis of medical drugs, determining the in vitro biological compatibility and important nanomedicine development parameters, such as IC10 and IC50, which are the inhibitory concentrations that induce a 10% and 50% reduction in cell viability, respectively. The evaluation can be assessed using ISO 10993 [44], an international standard for the biological evaluation of medical devices, which offers a study and assessment of the data already available from all sources along with the selection and use of additional tests if needed [45]. Preliminary biocompatibility tests, carried out with HDFn cells (Figure 5), showed biocompatibility in the range of 0.1–1.0 µg·mL^−1^ for the Ce6/NE and 0.1–1.5 µg·mL^−1^ for the unloaded/NE, with no statistically significant differences between the concentrations in the range, in accordance with ISO 10993-5 (cytotoxicity effect will only be considered for cell viability below 75%). The elevated cytotoxicity observed for the Ce6/NE at the higher concentrations can correlate to its increase in cell uptake, in contrast with the free Ce6. Comparing Figure 5A,B, it is possible to observe the cytotoxicity resulting only from the Ce6 (1.25 and 1.5 µg·mL^−1^), highlighting the amplification of properties using nanotechnology, achieving an excellent drug delivery at low concentrations. The free Ce6 also showed satisfactory results with no interference of DMSO. Previous studies assessing Chlorin e6 cytotoxicity in the absence of PDT light stimulation using fibroblast cells also showed the absence of toxicity in lower concentrations, making it a strong contender for use in biologically relevant protocols and upcoming research projects involving the assessment of photodynamic activity [46].

Over the past few years, efforts have increasingly focused on reducing the use of animals in research, especially in the evaluation of the safety of new chemicals and pharmaceuticals, following the 3R concept: replacement, refinement, and reduction of animals in research [47]. The 2D cytotoxicity assay is widely used; however, even though 2D culture still has its benefits, such as simplicity of method and low cost, the relevant biological environment is not mimicked with sufficient complexity [48]. The use of the chorionic allantoic membrane (CAM) model is an intermediate stage between in vitro and in vivo analysis, mimicking vascularized bio-barriers [49].

Biocompatibility and antiangiogenic studies were conducted using the HET-CAM assay, determining the irritation score and the tendency to reduce the vascular growth, respectively. As expected, the application of NaCl (negative control) produced no significant changes in the model, in contrast with the NaOH (positive control) where lysis, coagulation, and severe hemorrhage were observed. All the samples tested (Ce6/NE, unloaded/NE, and free Ce6) showed signs of biological compatibility (Figure 6), with the nanoemulsions not promoting additional toxicity, indicating potential safety for future applications [50,51]. Correlating with the monolayer model developed using HDFn cells, a broader range of biocompatibility can be identified. While the 2D model indicated compatibility up to 1.0 µg·mL^−1^ for the Ce6/NE, the HET-CAM model demonstrated biocompatibility up to 5.0 µg·mL^−1^, showing the advantages in the use of ex vivo models in drug development [52].

Tumor progression is intrinsically linked to angiogenesis, a complex biological process characterized by the formation of new blood vessels, required to sustain the high proliferative demands of cancer cells [53]. Although Ce6 is an extensively studied photosensitizer, its antiangiogenic action using ex vivo models is poorly addressed. Antiangiogenic studies were used to assess the Ce6/NE efficiency, showing the potential in vascular growth inhibition compared to the positive control in different concentrations (Figure 7). The results demonstrated the dose-dependent antiangiogenic activity of Ce6/NE, with the concentration at 2.5 µg·mL^−1^ showing the most significant inhibition rates and statistical relevance. Additionally, the potential of Ce6/NE to decrease the number of blood vessels formed was identified, especially when compared to methylene blue as the positive control. This showed the increased performance of Ce6 in nanoemulsions, corroborating its use in PDT protocols in skin cancer treatment. In this scenario, the nanotechnology mechanism is elucidated, showing a reduction in the effective concentration when compared with a previous study [2]. This allows achieving anticancer effects at lower concentrations of the active ingredient, resulting in the reduction in side effects and cytotoxicity.

Studies developed with Ce6-loaded nanosystems showed an increase in properties, as expected with the use of nanotechnology, presenting efficiency in PDT applied therapies. However, the systems showed high complexity with the use of high energy or organic solvents during the synthesis, aside from particle size within the range of 100–200 nm, which was strongly connected with PdI values. Combined, these factors lead to instability and an increase in the therapeutic dose in some cases [12,54,55]. A comparison with existing efforts highlights the simplicity of the Ce6/NE system, which manages to elevate the stability and improves the drug delivery of an FDA-approved anticancer pharmaceutical from the field of PDT.

## 4. Materials and Methods

### 4.1. Development of Ce6 Nanoemulsions Using the Low-Energy Method

The nanoemulsions were developed using the low-energy method, phase inversion composition (PIC), as described by [56] with modifications. Oil-in-water (O/W) nanoemulsions were prepared, with a composition of 2% (*w*/*w*) of isopropyl myristate (C_17_H_34_O_2_), 2% (*w*/*w*) surfactant mixture, Span 80% (C_24_H_44_O_6_) and Tween 20 (C_58_H_114_O_26_) in a proportion of 41.94% and 58.06%, respectively, 96% (*w*/*w*) ultrapure water, and Chlorin e6 at a concentration of 0.1 mg·mL^−1^. The lipid phase, including isopropyl myristate, surfactants, and Ce6, was heated to 50 °C, solubilizing the Ce6. Following homogenization, the ultrapure water was dripped in slowly with the aid of a syringe under constant agitation using a magnet stirrer. The formulation was homogenized for a further 1 min and then left to stand for 24 h, followed by subsequent analyses. Storage was carried out at 25 °C.

The real concentration (Rc) of Ce6 associated with the nanoemulsions was assessed using the theoretical concentration (Tc) of 0.1 mg·mL^−1^ and the calibration curve previously developed. Dimethyl sulfoxide (DMSO) was used as the organic solvent, as suggested by the manufacturer (MedKoo Biosciences, Durham, NC, USA). Linearity was developed as recommended by the FDA guidance [57] within the Ce6 concentration range (2–14 µg·mL^−1^) using a Thermo Scientific Genesys 10s UV/Vis dual-beam UV-Vis spectrophotometer (Waltham, MA, USA). The quantification was developed solubilizing the nanoemulsions in DMSO (40 °C–25 min) using an ultrasonic bath with subsequential absorption analysis, using the following equation to calculate the association efficiency of Ce6 in the nanosystem:

(1) Association efficiency%=1−(Tc−RcTc)×100

### 4.2. Characterization of Particle Size, Polydispersity Index (PdI), and Zeta Potential

The physicochemical analyses were performed following the standard operating procedures of the equipment Zetasizer^TM^, model Nano ZS90 Malvern (Malvern, UK), using a 1:1000 dilution, obtaining parameters of particle size, polydispersity index (PdI), and zeta potential for 180 days.

### 4.3. Atomic Force Microscopy

Atomic force microscopy allows the topographical surface of nanomaterials to be analyzed. Images of the Ce6/NE and unloaded/NE were taken at room temperature and relative humidity (10% and 25 °C) using a Bruker ^TM^ MultiMode8 atomic force microscope (Billerica, MA, USA) in Peak Force Tapping^TM^ mode. The images were obtained using the intermittent contact method, with a nominal spring constant of 0.4 N/m and a 2.0 nm tip end radius.

### 4.4. Fluorescence Characterization

#### 4.4.1. Two-Dimensional and Three-Dimensional Fluorescence Spectroscopy UV-Vis

The fluorescence emission spectra of the free Chlorin e6 and Ce6/NE were obtained as part of the spectroscopic characterization. For the fluorescence analyses, the samples were analyzed with fixed excitations at 4000 nm, an integration time of 0.3 s, excitation and emission slits of 10.0/10.0 nm, and an acquisition range of 450 to 900 nm, using a spectrofluorometer model RF-6000 (Shimadzu, Quioto, Japan). 

#### 4.4.2. Fluorescence Quantum Yield

The fluorescence quantum yield was determined by the relative method as described by [21]. The samples were prepared in water and PBS as the standard (Φ_f_ = 0.18) as previously described by [22] and evaluated using a UV-Vis spectrophotometer (Genesys 10S—Thermo Fisher Scientific, Waltham, MA, USA) in a range of 300–700 nm to determine the maximum absorption wavelength with an optical density of 0.1. A spectrofluorometer analysis was subsequently developed, with parameters previously determined by the absorbance analysis. The fluorescence quantum yields were then calculated using Equation (2) [21].

(2) $$\Phi_{f}=\Phi_{f}^{0}\times \frac{F_{s}}{F_{0}}\times \frac{A_{0}}{A_{s}}\times \frac{n_{s}^{2}}{n_{0}^{2}}$$

where Φ_f_ corresponds to the fluorescence quantum yield, F corresponds to the integrated fluorescence intensity, A corresponds to the absorbance at the excitation wavelength, and n corresponds to the refractive index of the solvent used. The subscript 0 refers to the reference, and the subscript s refers to the sample.

### 4.5. In Vitro Drug Release Analysis

#### 4.5.1. Solubility Analysis

The first solubility test was developed to identify the best release media for Ce6 under sink conditions. Four different types of release media were tested (PBS 6.4; PBS 6.4–15% Ethanol–2% Tween 80; PBS 6.4–10% DMSO–2% Tween 80; PBS 6.4–10% DMSO–5% Tween 80) with subsequent fluorescence analyses. The sink condition was calculated through the solubility analysis of Ce6, developed as described by [32]. An excessive quantity of free Ce6 (10 mg) was equilibrated with PBS 6.4–15% Ethanol–2% Tween 80 (500 µL) for 24 h under agitation. After this period, the sample was centrifuged at 800× *g* for 30 min and filtered in a filter syringe (filter syringe, 0.22 µm) and analyzed using the fluorescence method (n = 3). The sink condition was calculated maintaining 10% of the saturation concentration in the receptor media.

#### 4.5.2. In Vitro Release

The in vitro drug release of the Ce6/NE and free Ce6 was developed using a Franz diffusion cell system (Hanson Microette^TM^ HANSON 0700-1251, Chatsworth, CA, USA) with three modified Franz cells with a PVFD (polyvinylidene fluoride) hydrophilic membrane. The release medium containing PBS (pH 6.4), 15% Ethanol, and 2% Tween 80 was prepared as described by [16] considering the sink conditions of Chlorin e6 and the pH of skin cancer (close to 6.4). To increase the pharmaceutical application of the Ce6/NE, the nanoemulsion was prepared as thermoreversible polymer hydrogels (TRGs) using 23% Pluronic F-127 (Sigma-Aldrich, St. Louis, MO, USA). Briefly, 200 µL of the Ce6/NE formulation was applied on the top of PVFD membranes and placed between the donor compartment (6.5 mL) and the Franz cell receptor, keeping in contact with the receptor solution.

The experiment was developed at constant magnetic stirring (300 ± 0.2 rpm) and 37 ± 0.5 °C. During pre-establish times (10 min, 20 min, 30 min, 1 h, 2 h, 3 h, 4 h, 5 h, 6 h, 7 h, 8 h, and 24 h), aliquots of 1.0 mL were collected and analyzed using the fluorescence method with fixed excitation at 415 nm, excitation and emission slits of 10.0/10.0 nm, and an acquisition range of 600 to 700 nm (Shimadzu Spectrofluorometer model RF-6000). The aliquots were quantified using the calibration curve developed using the release media, with Chlorin e6 obtaining the real concentration of Ce6/NE released in time. The release kinetic was developed using the Sigma Plot 14 software, analyzing six conventional release models, Weibull, Korsmeyer–Peppas, Baker and Lonsdale, Higuchi, First Order, and Hixon and Crowell, obtaining data related to the specific parameter associated with each model.

### 4.6. Cell Culture

The Human, Neonatal (HDFn- ATCC^®^ PCS-201-010™) cell line was established in 75 cm^2^ culture flasks in Dulbecco’s Modified Eagle Medium (DMEM) supplemented with 10% fetal bovine serum. The cells were cultivated until confluence, followed by treatment with a 0.05% Gibco^®^ trypsin solution (Waltham, MA, USA) to release them from the monolayer. The trypsinization process was interrupted by the direct addition of supplemented culture medium and centrifuged at 300× *g* for 5 min. The cells were resuspended in fresh medium and seeded. The culture medium was completely renewed up to three times a week.

### 4.7. Cellular Uptake

The Human, Neonatal (HDFn-ATCC^®^ PCS-201-010™) cells were plated in 96-well plates (5 × 10^3^ cells/well) and maintained at 37 °C in an atmosphere containing 5% CO_2_ for 24 h. Subsequently, Ce6/NE was applied at concentrations of 0.1–1.5 µg·mL^−1^ and free Ce6 at 0.5 µg·mL^−1^ with internalization at three different times (3 h, 6 h, and 24). After the mentioned times, the respective plates were washed with PBS and quantified using the EnSpire^TM^ microplate reader (PerkinElmer, Shelton, CT, USA), where the fluorescence intensity was analyzed.

### 4.8. Metabolic Activity Assay—Resazurin Based

The cytotoxicity was assessed though the metabolic activity of the cells, using resazurin reduction. Resazurin is reduced to resofurin, a pink compound, by diaphorase enzymes as a report of metabolic activity of the cells, being detected by a simple fluorometric assay [58]. HDFn cells were seeded in 96-well plates (5 × 10^3^ cells/well) for 24 h followed by the addition of the treatments (Ce6/NE, unloaded/NE, and Chlorin e6). Free Chlorin e6 was solubilized with 0.25% DMSO and DMEM. After the 3 h of incubation, the wells were washed with PBS (phosphate buffered saline, pH = 7.4), and a solution with resazurin (10%) was added to the cells and incubated again for 3 h. The fluorescence was read using the excitation wavelength at 560 nm and emission wavelength at 590 nm on an EnSpireTM microplate reader (PerkinElmer, Shelton, CT, USA).

### 4.9. Ex Vivo Evaluation of the Biocompatibility and Antiangiogenic Activity of Ce6/NE Using the Hen’s Egg Chorioallantoic Membrane Assay (HET-CAM)

#### 4.9.1. Biocompatibility

Fertile eggs from Leghorn chickens were incubated horizontally in an automatic incubator for six days under strictly controlled temperature conditions (37.5 ± 0.5 °C) and relative humidity (between 55% and 65%). On the fourth day, a portion of the eggshell was carefully removed. The eggs were divided into eight experimental groups: negative control with 0.9% NaCl, positive control with 0.1 M NaOH, DMSO 0.5% control, unloaded/NE 10% (*v*/*v*), Ce6/NE at concentrations of 0.5 µg·mL^−1^, 5.0 µg·mL^−1^, and 10.0 µg·mL^−1^, and free Ce6 at 10.0 µg·mL^−1^ + 0.5% DMSO. On the sixth day, the eggs were prepared for testing. Initial images of the blood vessels were taken using a stereoscope before the compounds were applied. The blood vessels were observed for 300 s after the application of the compounds. At the end of this period, new images were captured to record changes in the vessels. Irritation was assessed based on the presence of hemorrhage, vascular lysis, and coagulation, with times recorded in seconds.

The semiquantitative HET-CAM test scale was used, ranging from (−) to (+++), as follows: (−), no reaction, with no signs of hemorrhage, lysis, or coagulation; (+), mild reaction, with discrete signs of hemorrhage, vascular lysis, or coagulation; (++), moderate reaction, with intermediate levels of hemorrhage, lysis, and coagulation; and (+++), severe reaction, with intense hemorrhage, lysis, and coagulation, as described by [59]. Embryo survival was monitored for up to 600 s after the compounds were applied.

#### 4.9.2. Antiangiogenic Assessment

Fertilized eggs were disinfected with 70% ethanol and incubated horizontally at 37 °C with 70% humidity for three days. On the fourth day of incubation, an opening was made in the eggshell to access the chorioallantoic membrane (CAM). Five experimental groups were established (n = 4): 0.9% NaCl as the negative control, 0.33 mg·mL^−1^ methylene blue as the positive control [60], and Ce6/Ne at three different concentrations (0.5, 1.0, and 2.5 µg/mL). Due to the rapid development of blood vessels during the first days of incubation, 300 μL of each solution was applied directly to the CAM. The openings were sealed with Parafilm^TM^ ( Bemis NA, Neenah, WI, USA) to prevent contamination, and the eggs were reincubated under the same conditions.

Forty-eight hours after application, i.e., on the sixth day, antiangiogenic activity was evaluated. The number of blood vessels in each group was observed, recorded, and analyzed using a stereomicroscope. The results were expressed as mean values with their respective standard deviations (SDs). Additionally, the percentage of inhibition was calculated for each compound using Equation (3):

(3) $$Inhibition\%=\left[1-\frac{mean\;number\;of\;vessels\;in\;the\;positive\;control\;group}{mean\;number\;of\;vessels\;in\;the\;treated\;group}\right]\times 100$$

### 4.10. Statistical Analysis

Statistical analysis was performed using a one-way analysis of variance (one-way ANOVA), with Tukey’s test (cytotoxicity, uptake, and HET-CAM) as the post-test for a multiple comparisons analysis between the groups (* *p* < 0.05; ** *p* < 0.001). All statistical analyses were carried out with n = 3.

## 5. Conclusions

A new therapeutic approach for cancer treatment combining Ce6 with nanotechnology demonstrated promising results, addressing biocompatibility and bioavailability in target sites. The nanoemulsions maintained submicron size and six-month stability, while enhancing fundamental properties, with the Fickian diffusion as transport release. Spectroscopic and fluorescence studies confirmed the preservation of Ce6’s original characteristics, supporting its use in PDT. In vitro 2D models showed biocompatibility and increased cell internalization, while ex vivo HET-CAM models revealed greater biocompatibility and antiangiogenic potential, highlighting differences in model responses and the applicable concentrations for anticancer studies. Overall, Ce6/NE emerges as a promising photosensitizer for PDT and cancer therapy, offering enhanced biocompatibility, targeted delivery, and effective therapeutic outcomes.

## Figures and Tables

**Table 1:** Dynamic light scattering (DLS) analyses. Data of day 1, day 180 (after synthesis), and the main average * for the particle size, PdI, and zeta potential of the Ce6/NE and unloaded/NE.

Samples	Particle Size Average (nm)		
	Day 1	Day 180	Average
Ce6/NE	67.55 ± 14.81	72.36 ± 5.94	62.07 ± 10.46
unloaded/NE	67.41 ± 15.69	62.12 ± 16.73	59.74 ± 10.77
	PdI		
	Day 1	Day 180	Average
Ce6/NE	0.182 ± 0.01	0.124 ± 0.08	0.178 ± 0.04
unloaded/NE	0.145 ± 0.06	0.180 ± 0.01	0.185 ± 0.03
	Zeta Potential		
	Day 1	Day 180	Average
Ce6/NE	−29.58 ± 2.05	−31.350 ± 4.08	−31.75 ± 3.41
unloaded/NE	−28.867 ± 1.18	−26.250 ± 6.29	−30.362 ± 4.52

* n = 2.

**Table 2:** Photophysical and spectroscopic data of Chlorin e6 in PBS 7.4 (standard) with Ce6 dimethyl sulfoxide (DMSO) as the polar aprotic solvent and Ce6/NE in water.

	Ce6 PBS 7.4	Ce6 DMSO	Ce6/NE
λ Soret max. (nm)	403	407	406
λ Excitation (nm)	407	407	407
λ Emission max. (nm)	662.4	669.0	668.6
Φ_f_	0.18 *	0.36	0.16

* Standard previously determined by ISAKAU et al. 2008 [22].

**Table 3:** Kinetics evaluation and assessment of in vitro Ce6 release (Ce6/NE and free Ce6) using different mathematical models.

	Global Adjustment		**Inflexion** Point Adjustment	
Mathematical Models	Ce6/NE	Free Ce6	Ce6/NE	Free Ce6
Weibull	R^2^ = 0.9834	R^2^ = 0.9786	R^2^ = 0.9818	R^2^ = 0.9898
	k = 4.6475	k = 4.3040	k = 46.6752	k = 3.9929
	b = 1.9344	b = 0.2180	b = 0.4796	b = 0.5353
Korsmeyer–Peppas	R^2^ = 0.8792	R^2^ = 0.6141	R^2^ = 0.9819	R^2^ = 0.6130
	k = 0.6132	k = 2.2074	k = 0.3082	k = 1.9373
	n = 0.3008	n = 0.1047	n = 0.4341	n = 0.1357
Baker and Lonsdale	R^2^ = 0.6226	R^2^ = −4.5063	R^2^ = 0.9706	R^2^ = −1.8142
	k = 5.3685 × 10^−7^	k = 6.5515 × 10^−7^	k = 7.9361 × 10^−7^	k = 1.3635 × 10^−6^
Higuchi	R^2^ = 0.6108	R^2^ = −4.5950	R^2^ = 0.9698	R^2^ = −1.8491
	k = 0.1773	k = 0.1952	k = 0.2166	k = 0.2828
First Order	R^2^ = −1.0314	R^2^ = −13.5953	R^2^ = 0.5453	R^2^ = −6.1378
	k = 5.5912 × 10^−5^	k = 5.7098 × 10^−5^	k = 0.0001	k = 0.0002
Hixon and Crowell	R^2^ = −1.0546	R^2^ = −13.6907	R^2^ = 0.5414	R^2^ = −6.1701
	k = 1.8326 × 10^−5^	k = 1.8690 × 10^−5^	k = 4.4599 × 10^−5^	k = 5.3941 × 10^−5^

## Data Availability

All the available data are reported in this work.
